# Supplementary material for: Structural filtering of functional data offered discriminative features for autism spectrum disorder
Source: PLoS One. 2022 Dec 6;17(12):e0277989. doi: 10.1371/journal.pone.0277989 (PMC9725140; doi:10.1371/journal.pone.0277989)
Supplement: Attachment — Submitted filename: Report on manuscript.pdf [file pone.0277989.s002.pdf]

Report on manuscript

Middle and High Frequency Bands of Structural Graph Provide Significant Functional Features for Autism Spectrum Disorder

by

Alireza Talesh Jafadideh , Babak Mohammadzadeh Asl

- **Overview of manuscript**

The authors investigate the possibility of using the topological filters to derive data with discriminative features between ASDs and TCs. They perform diffusion tensor imaging (DTI) to compute SC matrix. They obtain the topological filters using GFT tool and SC matrix. Using FC matrices, they derive the graph global metrics and triadic interaction metrics. They apply the topological filters on rfMRI data to obtain data in three independent frequency bands of structural graph. Some results are presented to compare the triadic interaction metrics between groups and between graph frequency bands.

- **Comments on text**

1. **New contribution**

GFBs in comparison to FFB can provide significant features under the same conditions.

The frequency bands of structural graph can reveal more intrinsic differences existing in functional data of ASDs and TCs, consequently, providing more information.

The significant differences between ASDs and TCs existed in the MFB and HFB.

2. **English**

The English in this paper is good.

Comments

The paper has sufficiently presented the research problem description, introduction, methods / techniques, results & discussion, concluding remarks and references.

However, the authors are pleased to consider the following suggestions to improve the quality of the article.

(a) I suggest the title be rephrased to be shorter but at the same time to capture the content and its significance of this research.

(b) “The general linear model (GLM) was applied with age, diagnosis, and site variables as between covariate” Did the others try other statistical modeling which is more accurate in most cases such as generalized linear mixed models (GLMM)? The authors need to provide more evidence on this model selection.

(c) The authors mentioned “The non-parametric permutation testing with 400 repetitions.” Could they verify how they obtained this number? Based on which analysis they concluded that “ This process was repeated 400 times to obtain a distribution of measure difference”.

(d) The authors need to provide more details on the “statistical comparison” test they have used. The same for global efficiency.
(e) The authors should take into account briefly answering this question in the conclusion part “What is the implication of your results in the related application area?”
(f) The authors may need to have more discussion on the connection between “abnormal connectivity in the ASD brains” as they explained in other studies in the introduction part and their results.
(g) For “Limitations and future directions”, the authors also can think about other factors since “Individuals with ASD show enormous heterogeneity depending on age, gender, intellectual ability, genetic factor, and environmental risk factor (Lenroot RK et al., 2013).” Since “Studies regarding these affective factors will bring more consistent data and improve understanding of neurobiological mechanisms of ASD (Sungji Ha et al., 2015).”

- ## Recommendation

After all the revisions, I can recommend acceptance of this paper.
