# Supplementary material for: Structural filtering of functional data offered discriminative features for autism spectrum disorder
Source: PLoS One. 2022 Dec 6;17(12):e0277989. doi: 10.1371/journal.pone.0277989 (PMC9725140; doi:10.1371/journal.pone.0277989)
Supplement: Attachment — Submitted filename: ReviewComments.pdf [file pone.0277989.s003.pdf]

Reviewer comments for the article Middle and High Frequency Bands of Structural Graph Provide Significant Functional Features for Autism Spectrum Disorder

In this article, the authors investigate "Can filtering the functional data through the frequency bands of structural graph provide data with valuable features for Austism Spectrum Disorder?" The goal is to see if there are any interesting discriminative features which are otherwise not present in an unfiltered data in the ASD and Typical Controls (TC). The study uses standard graph metrics and a metric based on functional triadic interactions and the results show differences in the ASD and TC at different frequency bands. This study on structural filtering of functional data for feature extraction is interesting to read. The results indicate the usefulness of structural graph frequency bands to evaluate functional data and allows to uncover features which are hitherto hidden is an important finding from this study. However, there is lack of reasoning or details to the methodology adopted and why this approach should be attempted? The paper does not address motivation behind the proposed method and gap in literature to support the same.

These issues have to be well addressed in both the introduction and methodology section. Besides, the authors should present the how stable the results are by varying the range of Low, Medium and High frequency bands instead of dividing the frequency band almost equally into 33, 34 and 33 modes. From my own prespective, the study cannot be accepted as long as the following comments have been addressed and the respective replies and clarifications have been incorporated into the revised manuscript.

1. It will be interesting to see how the stable the results are to the choice of the range chosen for assigning the frequency bands? The authors can provide explanations or comments on why the frequency bands were divided almost equally? Or plot the diagonal filtering matrix to see if there is any sharp transition between the modes?

2. The numerical implementation of the method is not explained in detail. How are the times series data of 155 time points is handled in the graph metrics computation in section 2.6.1? How many triads in total are present in the ASD and TC? Is there any thresholding implemented on the triads? Please explain in terms of the softwares used if any, the size of the data used and computation time etc.

3. Although the authors claim using one modality (FC) to employ thier GSP as a future work, it would be better to mention why SC was chosen for computing the frequency bands and why it was not directly done on the FC of rfMRI in the introduction?

4. It is unclear how the time series data is handled while computing the Global metrics of the graph? Please mention if its a simple averaging of time series?

5. Can this method identify or localize specific brain regions (ROIs) that may cause these significantly discriminating features? Since triadic interactions are quantified in this paper, Is it possible extract and plot brain regions where energy (Un) is maximum?

Minor comments to be addressed are

- A figure that explains the methodogy as a flowchart or pipeline can be added to follow the paper more clearly.
- Although the observed differences are explained in the discussion section. The motivation behind choice of graph measures such as assortivity, clustering coefficients and efficency can be included in the introduction with relevant literature. The relevance of these measures in the context of ASD needs to be explained?
- The frequency of triads notation should be written as subscripts in section 2.6.2 Triadic interactions and their metrics and also in other places to be consistent.
- Figure 1 caption is in italics change it to normal font style. Also the caption can be more specific to what the figure attempts to illustrate?
- The plot of energy distributions as log distribution for the AUD and TC at different frequency bands may provide a better representation of the energy.
