# Supplementary material for: Structural filtering of functional data offered discriminative features for autism spectrum disorder
Source: PLoS One. 2022 Dec 6;17(12):e0277989. doi: 10.1371/journal.pone.0277989 (PMC9725140; doi:10.1371/journal.pone.0277989)
Supplement: Attachment — Submitted filename: Response to Reviewers.pdf [file pone.0277989.s004.pdf]

Dear reviewer, we are thankful for your time, consideration, and constructive comments. In this document, we have provided our answers to your comments, point by point, and referred to the parts of the paper where the comments have been applied.

In the following, our answers to the comments are provided (line numbers are on the right side of the manuscript).

Reviewer 1

1. I suggest the title be rephrased to be shorter but at the same time to capture the content and its significance of this research.

In the new version of manuscript, this comment has been applied.

2. "The general linear model (GLM) was applied with age, diagnosis, and site variables as between covariate" Did the others try other statistical modeling which is more accurate in most cases such as generalized linear mixed models (GLMM)? The authors need to provide more evidence on this model selection.

The GraphVar software uses GLM tool for statistical analysis. Hence, at the first, the GLM was considered to be used for between groups comparison. However, we used a more accurate approach of GLM i.e., stepwise GLM:

[The model for analyzing the effect of the diagnosis variable is defined as

$$\boldsymbol{y} = \beta_0 + \beta_{age}\boldsymbol{x}_{age} + \beta_{diagnosis}\boldsymbol{x}_{diagnosis} + \beta_{age\_diagnosis}\boldsymbol{x}_{age}\boldsymbol{x}_{diagnosis} + \boldsymbol{\epsilon} \qquad (12)$$

where **y** is a vector containing both ASD and TC measures from which the site effect was regressed out. This model was performed using the command "stepwiseglm" of MATLAB software with the constant model as the starting model, interaction model as the upper model, and deviance as the criterion for adding or removing terms [10].] [lines 18-23 of page 14]

This model has also been used by Mash and colleagues and Rashid and colleagues in autism studies [References 10 and 12 of manuscript].

The *p-values* were corrected by permutation test with 1000 repetitions. Thus, the results of *p-values* can be trustable because of using permutation test and without considering any assumption about distribution.

3. The authors mentioned "The non-parametric permutation testing with 400 repetitions." Could they verify how they obtained this number? Based on which analysis they concluded that "This process was repeated 400 times to obtain a distribution of measure difference".

In the new version of manuscript, the results are modified for permutation testing with 1000 repetition. There were seen no differences in terms of changing significant results to not significant and vice versa. The modification has been applied on the results of Table 2, Table 4, Figure 3, Figure 4, and texts of result section.

4. The authors need to provide more details on the "statistical comparison" test they have used. The same for global efficiency.

In the new version of manuscript, the GLM analysis has been explained in more details:

[The model for analyzing the effect of the diagnosis variable is defined as

$$\boldsymbol{y} = \beta_0 + \beta_{age}\boldsymbol{x}_{age} + \beta_{diagnosis}\boldsymbol{x}_{diagnosis} + \beta_{age\_diagnosis}\boldsymbol{x}_{age}\boldsymbol{x}_{diagnosis} + \boldsymbol{\epsilon} \qquad (12)$$

where **y** is a vector containing both ASD and TC measures from which the site effect was regressed out. This model was performed using the command "stepwiseglm" of MATLAB software with the constant model as the starting model, interaction model as the upper model, and deviance as the criterion for adding or removing terms [10].] [lines 18-23 of page 14]

In the new version of manuscript, the Wilcoxon rank-sum test was used instead of t-test:

[For a given group, the comparison between GFBs was performed using the Wilcoxon rank-sum test (Mann and Whitney, 1947) as a nonparametric test] [lines 1-2 of page 15]

The results did not show differences in terms of changing significant results to not significant and vice versa. The results are provided in the texts of result section and in the Figures 3 and 4.

Also, all the *p-values* were corrected by permutation test with 1000 repetitions. Thus, the results of *p-values* can be trustable because of using permutation test and without considering any assumption about distribution.

The following explanation has been added to the manuscript for the global efficiency of graph:

[The **efficiency** can be formulated as

$$\boldsymbol{Efficiency} = \left(\sum_{i\neq j} 1/l_{ij}\right)/M(M-1) \quad (5)$$

where l_{ij} is the shortest path length between i[th] and j[th] ROIs and M represents total number of ROIs.] [lines 17-19 of page 12]

5. The authors should take into account briefly answering this question in the conclusion part "What is the implication of your results in the related application area?"

In the new version of manuscript, this comment has been applied.

[The implication of these results is that analyzing functional features in structural frequency bands can introduce discriminative features which were not discriminative in unfiltered functional data. Therefore, employing structural filters may provide a new avenue for extracting features which can be considered as candidate biomarkers for ASD.] [lines 9-12 of page 26]

6. The authors may need to have more discussion on the connection between "abnormal connectivity in the ASD brains" as they explained in other studies in the introduction part and their results.

In the new version of manuscript, this comment has been applied.

[In the MFB, the clustering coefficient of ASDs was significantly larger than TCs. Thus, the results of the clustering coefficient in the MFB may show a decrease of brain functional specialization in ASDs due to an abundance of functional connections (increased clusters of local connections in ASDs)] [lines 16-18 of page 20]

[In the MFB and HFB, the connection strength of ASDs was significantly larger than TCs. This result may show the over-connectivity in ASDs compared to TCs in the MFB and HFB. The over-connectivity has been reported in many ASD studies (Ye et al., 2014; Supekar et al., 2013; Hull et al., 2016; Di Martino et al., 2013).] [lines 19-21 of page 20]

[The clustering coefficient and strength results indicated the local over-connectivity and greater local efficiency in ASDs. The efficiency and strength results suggest a pattern of global overconnectivity. All of these indicate the disruption of balance between network segregation (revealed by clustering coefficient and strength) and integration (revealed by efficiency and strength) in ASDs. This conclusion is in line with the results of SWN and SWP. Several studies reported disruption between segregation and integration (Barttfeld et al., 2011; Barttfeld et al., 2012; Ye et al., 2014; Soma et al., 2021).] [lines 1-5 of page 21]

[One of the results of this study was local functional over-connectivity in ASDs. There are some pathophysiological evidence and findings that support this result. One of the findings is the imbalance in excitatory/inhibitory neural activity (Yenkoyan et al., 2017). Diminished GABAergic function is hypothesized to disrupt the excitatory/inhibitory balance at the neuronal level which in turn, leads to mostly over-connectivity of ROIs (networks) in ASD (Pizzarelli and Cherubini, 2013). Another pathophysiological finding is that people with autism do not undergo normal pruning during childhood and adolescence (Tang et al., 2014). A synapse allows for neural communication between cells. When there are too many synaptic connections, the brain goes through a process of cutting down; known as synaptic pruning (cutting out the non-functional, unnecessary neurons to increase the power of the working ones). Studies in neural density have found a higher number of neurons among autistic individuals which is due to a slowdown in a normal brain "pruning" process during development (Tang et al., 2014; Courchesne et al., 2014; Casanova et al., 2002)]. It has been proposed that an excess of neurons causes local functional over-connectivity (Courchesne et al., 2007; Keown et al., 2013). Thus, our findings may reflect an immature connectivity pattern in ASDs.] [lines 6-15 of page 21]

7. For "Limitations and future directions", the authors also can think about other factors since "Individuals with ASD show enormous heterogeneity depending on age, gender, intellectual ability, genetic factor, and environmental risk factor (Lenroot RK et al., 2013)." Since "Studies regarding these affective factors will bring more consistent data and improve understanding of neurobiological mechanisms of ASD (Sungji Ha et al., 2015)."

In the new version of manuscript, this comment has been applied.

[Overall, enormous heterogeneity of ASD depends not only on age and gender but also on intellectual ability, genetic factor, and environmental risk factor (Lenroot and Yeung, 2013). Hence, if future studies take into account these affective factors, they will bring more consistent data and repeatable results and improve understanding of neurobiological mechanisms of ASD (Ha et al., 2015).] [lines 1-3 of page 25]

Reviewer 2

This paper is overall coherent. The introduction provides reasonable background from the literature on the analytical pipeline used in the study. However,

1. Methodological components need more justification rather than stating what they are and so and so paper used them. For instance, there are quite a few complex network measures (Rubinov and Sporns, 2010), or global metric of graph as the paper refers to them, that one can use. Why were assortativity, clustering coefficient, efficiency, radius, diameter, strength, SWN, and small-world propensity (SWP) the only ones studied?

We have added the following explanations to the introduction section:

[Many of autism studies have described patterns of under- or over-connectivity (Di Martino et al., 2013; Reday E et al., 2013; Hull et al., 2016) which can be led to abnormal segregation (independent processing in specialized subsystems) and integration (global cooperation between different subsystems) of resting-state brain (King et al., 2019; Hong et al., 2019; Henry et al., 2018). In this study, metrics of graph were used to assess the differences of functional integration and segregation between TC and ASD. Some studies have shown good to excellent repeatability for global metrics, while for local metrics it was more variable and some metrics were found to have locally poor repeatability (Andreotti et al., 2014; Cao et al., 2014; Niu et al., 2013; Telesford et al., 2010). As a result, the global metrics of graph were used in this study. In some studies, the **clustering coefficient** (metric for segregation analysis) and **efficiency** (metric for integration analysis) have been reported as the most reproducible metrics of graph (Niu et al., 2013; Telesford et al., 2010). The **strength** is another metric for studying over/under-connectivity in autism (Ye et al., 2014; Supekar et al., 2013; Hull et al., 2016). Another global metric is **assortativity coefficient**. Generally, an assortative network is robust against selective ROI failure, and this accelerates the spread of information generated by high-degree ROIs. The excessive assortativity led to poor network performance (Murakami et al., 2017). Itahashi and colleagues (Itahashi et al., 2014) reported reduced assortativity in ASD. This finding is consistent with the view that network organization in the ASD brain shifts toward randomization compared to that in the TC brain. In addition to assortativity, the decreased clustering coefficient and increased efficiency also inform about increased of brain network randomness (Itahashi et al., 2014; Rudie et al., 2013). The more/less values of **radius** and **diameter** metrics can show more/less correlated neural activity in spatially distributed ROIs in ASD group compared to TCs (Malaia et al., 2016). The **SWN** and small-world propensity (**SWP**) are other well-known metrics of graph tending to display a balance between segregation and integration (Bassett and Bullmore, 2017; Fornito et al., 2016). In this study, the metrics written in bold face were employed to see if graph frequency bands can reveal abnormality in terms of segregation and integration or under/over-connectivity or increased/decreased randomness of ASD brain. Our aim is not to use all of the global metrics of graph. These aforementioned metrics can be enough for answering to our questions raised in sixth paragraph of this section.] [lines 5-23 of page 5].

Besides these explanations, it is better to explain another reason for working with studied metrics. The global metrics of graph were computed using the GraphVar software. We could only select three or four global metrics. However, computation of more global metrics had no computational or other costs. Hence, we selected as many metrics, as possible. Hence, the assortativity, diameter, and radius were also selected and computed. The density metric was equal between groups and GFBs (40% of connections were preserved through PT and so the density was the same for all groups and GFBs) and the characteristic path

length was inversely related to efficiency. Thus, these metrics were not studied. All other global metrics of GraphVar were selected. The modularity was investigated through community structure analysis (section 2 of supplementary file). Instead of only reporting metrics with significant results, we thought it was better to also report the results of metrics having no significant results between ASD and TC groups. Thus, another reason was easy analysis of more global metrics by GraphVar.

2. Triadic interactions are an interesting piece of the study. But why use the approach of Moradimanesh et al. and no other hyper graph approaches?

Our main goal was to investigate if the frequency bands of structural graph were informative? Or more informative than unfiltered rfMRI data sometimes? To do this investigation, the ordinary graph metrics were used. The ordinary graph was well-known for us and many researchers and it was used in many studies. Therefore, it could be a good choice for our investigation purpose. The ordinary graph uses dyadic interaction of brain network. The triadic interactions were recently studied in an ASD study by Moradimanesh and colleagues. Their study increased the level of interaction from 2 to 3. Also, they reported interesting results and their approach was new. Fortunately, we got familiar with their paper and research and because of aforementioned advantages, we thought that triadic interactions would be an interesting and a good choice for our analysis.

To be honest, unfortunately, we did not know about hyper graph approaches and we were not familiar with them. Else, we would have definitely used these approaches beside ordinary graph. However, we have listed it as a future work. Also, in the "*Limitations and future works*" subsection, we have proposed this idea.

[Overall, in this study, the connectivity matrices of rfMRI data were analyzed through ordinary graph metrics. In an ordinary graph, an edge connects exactly two vertices. For future work, the hyper graph approaches can be used to compare filtered and unfiltered rfMRI data and to compare ordinary graphs versus hyper graphs in different frequency bands of structural graph. A hypergraph is a generalization of a graph in which an edge can join any number of vertices (Berge and Minieka, 1973).] [lines 19-22 of page 25]

3. Statistical analysis consists of GLM and t-test, both of which rely on stringent distributional assumptions. How do we know that these assumptions are not violated by the complex neuroimaging data used in the study?

The GraphVar software uses GLM tool for statistical analysis. Hence, at the first, the GLM was considered to be used for between groups comparison. However, we used a more accurate approach of GLM i.e., stepwise GLM:

[The model for analyzing the effect of the diagnosis variable is defined as

$$\boldsymbol{y} = \beta_0 + \beta_{age}\boldsymbol{x}_{age} + \beta_{diagnosis}\boldsymbol{x}_{diagnosis} + \beta_{age\_diagnosis}\boldsymbol{x}_{age}\boldsymbol{x}_{diagnosis} + \boldsymbol{\epsilon} \qquad (12)$$

where **y** is a vector containing both ASD and TC measures from which the site effect was regressed out. This model was performed using the command "stepwiseglm" of MATLAB software with the constant

model as the starting model, interaction model as the upper model, and deviance as the criterion for adding or removing terms [10].] [lines 18-23 of page 14]

This model has also been used by Mash and colleagues and Rashid and colleagues in autism studies (References 10 and 12 of manuscript).

In the new version of manuscript, the Wilcoxon rank-sum test was used instead of t-test:

[For a given group, the comparison between GFBs was performed using the Wilcoxon rank-sum test (Mann and Whitney, 1947) as a nonparametric test] [lines 1-2 of page 15]

The results did not show differences in terms of changing significant results to not significant and vice versa. The results are provided in the texts of result section and in the Figures 3 and 4.

All the *p-values* were corrected by permutation test with 1000 repetitions. Thus, the results of *p-values* can be trustable because of using permutation test and without considering any assumption about distribution.

Reviewer 3

1. It will be interesting to see how the stable the results are to the choice of the range chosen for assigning the frequency bands? The authors can provide explanations or comments on why the frequency bands were divided almost equally? Or plot the diagonal filtering matrix to see if there is any sharp transition between the modes?

At the first, by plotting the normalized eigenvalues of Laplacian matrix (more explanation is given in Figure R3- 1), the first and the last 20 eigenvectors of Laplacian matrix were selected as frequency modes of LFB and MFB, respectively. However, there were seen no significant results. The simplest way that one can propose for division is dividing the frequency modes to an equal size. As a result, we taken into account equal size division and significant results were obtained. Such division offered results for graph metrics, triadic interaction metrics and community structure analysis which were in line with each other and confirmed each other. Thus, such in line results provided reliability for us. Also, the results of FA200 confirmed that equal division is trustable.

For sensitivity analysis:

[In this study, equal number of frequency modes were considered to make LFB, MFB, and HFB. This division was token because it was the simplest way and the first thing that comes to mind. Such division offered results for graph metrics, triadic interaction metrics and community structure analysis which were in line with each other and confirmed each other. Thus, such in line results provided reliability for us. Also, the results of FA200 confirmed that equal division is trustable. However, sensitivity analysis may be useful to see how the stable the results are to the choice of the range chosen for assigning the frequency modes. For this analysis, the between group results of triadic interaction metrics were studied. The frequency modes of LFB, MFB, and HFB were considered as $[v_1, v_2, \dots, v_L]$, $[v_{ML}, v_2, \dots, v_{MH}]$, and $[v_H, v_{H+1}, \dots, v_{100}]$, respectively. In this study, the values of L, ML, MH, and H were 33, 34, 67, and 68, respectively. To investigate the sensitivity, the values of L, ML, MH, and H were decreased and increased by 1, 2, 3, 4, and 5. Thus, the L of v_L was changed from 28 to 38 and there were seen no significant alternations in the results of LFB, i.e., changing from not significant to significant didn't happen. Also, by decreasing and increasing of ML, MH, and H there were seen no meaningful alternations in terms of changing significant results to not significant and vice versa. The behavior in LFB did not change even for $L = 20 \; or \; 40$. However, increased the H by 10 led to changing of Un and $|T_3|$ results from significant to not significant (t = -1.09, p = 0.16; t = 1.6, p = 0.075). Decreased H by 10 led to no differences in significant results. Also, decreased and increased of ML and MH by 10 led to no differences in significant results. These results show the less sensitivity of our findings to the choice of the range chosen for assigning the frequency modes.] [lines 1-16 of page 24]

For graph metrics, for attaining the results related to each number of frequency modes, it was needed to upload the filtered rfMRI to the GraphVar software. Also, the computation time of SWN metric was several hours for each filtered data. Thus, it took a lot time to obtain results of graph metrics for each number of frequency modes (for example, for 25 or 26 or 27 or … frequency modes of LFB, the uploading filtered rfMRI data and graph metrics computation were performed separately). Hence, for sensitivity analysis, only the triadic metrics were used.

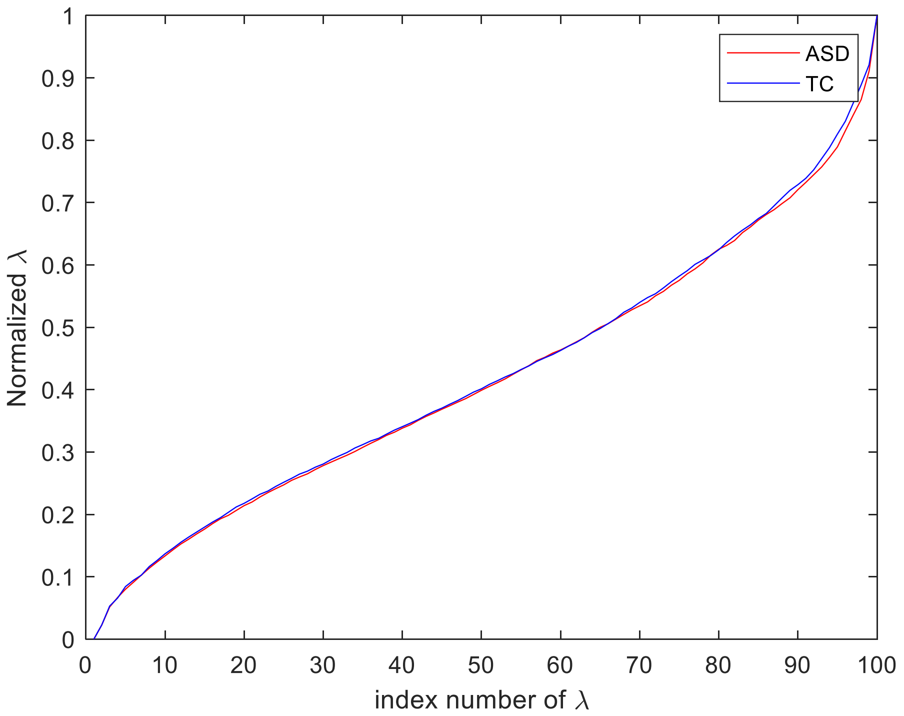

Figure R3- 1- The normalized eigenvalues of Laplacian matrix averaged over subjects. For each subject, the eigenvalues of Laplacian matrix were normalized by dividing each eigenvalue to maximum eigenvalue of that matrix. Therefore, each subject had 100 (the number of ROIs) normalized eigenvalues. For each group, the normalized eigenvalues were averaged over subjects of that group to obtain the final normalized eigenvalues. These normalized averaged eigenvalues are plotted for ASD and TC groups by red and blue color lines, respectively. Eigenvalue is indicated by character λ.

2. The numerical implementation of the method is not explained in detail. How are the times series data of 155 time points is handled in the graph metrics computation in section 2.6.1? How many triads in total are present in the ASD and TC? Is there any thresholding implemented on the triads? Please explain in terms of the softwares used if any, the size of the data used and computation time etc.

Always, the graph metrics are computed using the connectivity matrices. In this study, the connectivity matrices were computed by tHOFC method (subsection 2.5). For handling time points:

[Calculation of connectivity matrices was performed using the GraphVar software (Kruschwitz et al., 2015). The rfMRI data of LFB, MFB, HFB, and FFB were separately loaded in this software. Then, calculation of tHOFC was performed for studied subjects. All 155 time points of ROIs were used to compute one tHOFC matrix for each subject. For calculating the studied metrics, we did not deal with 155 time points of ROIs, instead, we dealt with one sparse connectivity matrix (sparse tHOFC matrix).] [lines 14-17 of page 11].

In the previous and current versions of manuscript, we have explained that:

[The sparse tHOFC matrices were used for computation of studied metrics. The global metrics of graph were computed by GraphVar software (Kruschwitz et al., 2015). The analysis of triadic interactions was performed by personal codes.] [lines 19-20 of page 11]

We have mentioned in Table 1 that the data of 53 ASD and 45 TC subjects was used in this study. Also, in subsection 2.3 and subsubsection 2.2.1, we have mentioned that one ASD subject of NYU1 and one TC subjects of SDSU were removed from analysis, respectively. In the subsection 2.3, we have explained that the Schaefer atlas (Schaefer et al., 2018) with 100 ROIs was employed for brain parcellation. Thus, the dimension of connectivity matrices is 100*100. However, in the new version of manuscript, we have provided all of this information in one place:

[The scenarios of this study were implemented using data of 52 ASD patients and 44 TC subjects of NYU1 and SDSU datasets. The dimension of tHOFC connectivity matrices was 100*100 and 200*200 for Schaefer atlas with 100 ROIs and 200 ROIs, respectively.] [lines 1-3 of page 12]

[There was no thresholding on the triads. The thresholding was only applied on the tHOFC matrix for attaining a sparse matrix. Then, the triads were computed using sparse tHOFC matrices.] [lines 20-22 of page 11]

For the number of triads:

If the connectivity matrices were full (no sparse matrices), then the number of triads was the same for all subjects and depended on only to the number of ROIs. However, in this study, the connectivity matrices were sparse. Although the number of connections were the same for all subjects in sparse matrices, the connections can be different (for example, one subject had no connection between ROI1 and ROI2 and had connection between ROI1 and ROI3 whereas another one had connection between ROI1 and ROI2 and had no connection between ROI1 and ROI3). As a results, the number of triads can be different between subjects (Please see Figure R3-2). Hence, in the new version of manuscript, the average of the number of triads for ASD and TC groups have been reported (averaged across subjects):

[On average, the total number of triads in the (LFB, MFB, HFB, FFB) were (86648,86002,86875, 88310) and (86043,85333,85950,88598) for ASDs and TCs, respectively.] [lines 5-6 of page 19]

For computation time:

[All the graph metrics (except SWN) were computed together in the GraphVar software. The computation time of these processes was about 2 minutes. The mentioned processes were repeated for SWN with computation time of about 450 minutes. The computation time of all triad metrics (together) was about 1 minute. These times are times needed to compute metrics for all studied subjects. All the computation was performed using a PC Core i7-8700k @ 3.7GHz.] [lines 5-8 of page 23]

The computation of SWN needs to generate null model (for more explanations, please read the book entitled as “Fundamentals of Brain Network Analysis”). The settings in GraphVar for SWN computation is shown in Figure R3-3. The default number for generation of random network was 1000 in GraphVar. As a result, the computation of SWN took several hours.

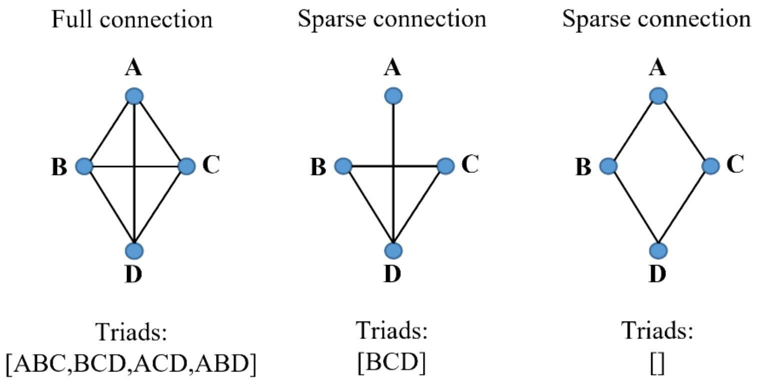

Figure R3- 2- Simple representation of the effect of sparsity on the number of triads. The left side of figure represents full connection for four ROIs including A, B, C, and D. the number of triads is 4. The middle and right side of figure represents the sparse version of left side. The number of connections of sparse versions are the same. However, the preserved connections (not removed during sparsity process) are different. As a result, the number of triads for the middle and right side are different.

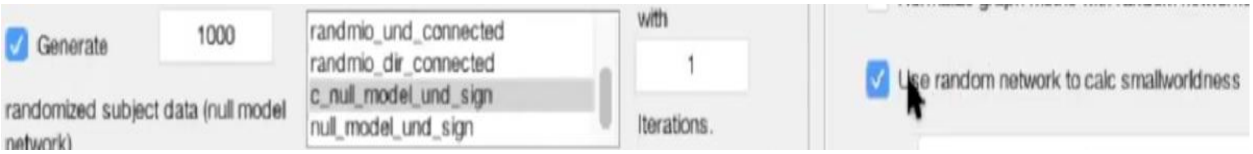

Figure R3- 3- The setting in GraphVar for computation of SWN.

3. Although the authors claim using one modality (FC) to employ their GSP as a future work, it would be better to mention why SC was chosen for computing the frequency bands and why it was not directly done on the FC of rfMRI in the introduction?

The following paragraph has been added to the introduction:

[The graph of GSP is considered as topology (structure) of studied system (herein is brain). It is expected that topology becomes stable during the time. The stability of SC is much more than FC. As a result, the structural connectivity (and not the functional connectivity) is used to model the graph of GSP in many neuroimaging studies (Brahim and Farrugia, 2020; Itani and Thanou, 2021; Jafadideh and Asl, 2021). Hence, in this study, the SC was used to model the graph of GSP.] [lines 1-4 of page 5]

Also, the following explanations have been added to the "Limitations and future works":

[However, as explained in the introduction section, it is convenient to have more stable topology, as possible. Hence, it is needed to sparse FC and preserve only the strongest connections. The stability of stronger functional connections is more than weaker ones (Thompson and Fransson, 2015; Mash et al., 2019).] [lines 6-8 of page 25]

4. It is unclear how the time series data is handled while computing the Global metrics of the graph? Please mention if its a simple averaging of time series?

Always, the graph metrics are computed using the connectivity matrices. In this study, the connectivity matrices were computed by tHOFC method (subsection 2.5). For handling time points:

[Calculation of connectivity matrices was performed using the GraphVar software (Kruschwitz et al., 2015). The rfMRI data of LFB, MFB, HFB, and FFB were separately loaded in this software. Then, calculation of tHOFC was performed for studied subjects. All 155 time points of ROIs were used to compute one tHOFC matrix for each subject. For calculating the studied metrics, we did not deal with 155 time points of ROIs, instead, we dealt with one sparse connectivity matrix (sparse tHOFC matrix).] [lines 14-17 of page 11].

Also, we have add:

[It should be mentioned that the size of LFB, MFB, HFB, and FFB data matrices are equal to $N \times 155$ in this study. The N is the number of ROIs.] [lines 19-20 of page 10].

5. Can this method identify or localize specific brain regions (ROIs) that may cause these significantly discriminating features? Since triadic interactions are quantified in this paper, Is it possible extract and plot brain regions where energy (Un) is maximum?

This method (filtering the rfMRI data in frequency bands of structural graph) only filters the data. Thus, it only changes the data and does not localize specific brain regions. Even the size of data does not change (the filtered data has the same size of unfiltered data (100*155 in our study)). The post analysis of these filtered data may reveal significant differences between ASD and TC groups at the ROI or entire brain level (differences which were not revealed using unfiltered data). The post analyses of our study were global metrics of graph and metrics of triadic interactions. Thus, for localizing brain ROIs, the used filtering technique can not be employed. However, for post analysis, local metrics of graph can be employed to reveal significant differences at the ROI level.

In this study, global metrics of graph were selected and computed in the GraphVar software. Also, the U(N) and triadic metrics were global. These metrics describe the properties governing the entire brain (all the ROIs).

6. A figure that explains the methodogy as a flowchart or pipeline can be added to follow the paper more clearly.

In the new version of manuscript, this comment has been applied. Overview of pipeline steps and study design has been given in Fig 1 of new version of manuscript.

7. Although the observed differences are explained in the discussion section. The motivation behind choice of graph measures such as assortivity, clustering coefficients and efficency can be included in the introduction with relevant literature. The relevance of these measures in the context of ASD needs to be explained.

We have added the following explanations to the introduction section:

[Many of autism studies have described patterns of under- or over-connectivity (Di Martino et al., 2013; Reday E et al., 2013; Hull et al., 2016) which can be led to abnormal segregation (independent processing

in specialized subsystems) and integration (global cooperation between different subsystems) of resting-state brain (King et al., 2019; Hong et al., 2019; Henry et al., 2018). In this study, metrics of graph were used to assess the differences of functional integration and segregation between TC and ASD. Some studies have shown good to excellent repeatability for global metrics, while for local metrics it was more variable and some metrics were found to have locally poor repeatability (Andreotti et al., 2014; Cao et al., 2014; Niu et al., 2013; Telesford et al., 2010). As a result, the global metrics of graph were used in this study. In some studies, the **clustering coefficient** (metric for segregation analysis) and **efficiency** (metric for integration analysis) have been reported as the most reproducible metrics of graph (Niu et al., 2013; Telesford et al., 2010). The **strength** is another metric for studying over/under-connectivity in autism (Ye et al., 2014; Supekar et al., 2013; Hull et al., 2016). Another global metric is **assortativity coefficient**. Generally, an assortative network is robust against selective ROI failure, and this accelerates the spread of information generated by high-degree ROIs. The excessive assortativity led to poor network performance (Murakami et al., 2017). Itahashi and colleagues (Itahashi et al., 2014) reported reduced assortativity in ASD. This finding is consistent with the view that network organization in the ASD brain shifts toward randomization compared to that in the TC brain. In addition to assortativity, the decreased clustering coefficient and increased efficiency also inform about increased of brain network randomness (Itahashi et al., 2014; Rudie et al., 2013). The more/less values of **radius** and **diameter** metrics can show more/less correlated neural activity in spatially distributed ROIs in ASD group compared to TCs (Malaia et al., 2016). The **SWN** and small-world propensity (**SWP**) are other well-known metrics of graph tending to display a balance between segregation and integration (Bassett and Bullmore, 2017; Fornito et al., 2016). In this study, the metrics written in bold face were employed to see if graph frequency bands can reveal abnormality in terms of segregation and integration or under/over-connectivity or increased/decreased randomness of ASD brain. Our aim is not to use all of the global metrics of graph. These aforementioned metrics can be enough for answering to our questions raised in sixth paragraph of this section.] [lines 5-23 of page 5].

Besides these explanations, it is better to explain another reason for working with studied metrics. The global metrics of graph were computed using the GraphVar software. We could only select three or four global metrics. However, computation of more global metrics had no computational or other costs. Hence, we selected as many metrics, as possible. Hence, the assortativity, diameter, and radius were also selected and computed. The density metric was equal between groups and GFBs (40% of connections were preserved through PT and so the density was the same for all groups and GFBs) and the characteristic path length was inversely related to efficiency. Thus, these metrics were not studied. All other global metrics of GraphVar were selected. The modularity was investigated through community structure analysis (section 2 of supplementary file). Instead of only reporting metrics with significant results, we thought it was better to also report the results of metrics having no significant results between ASD and TC groups. Thus, another reason was easy analysis of more global metrics by GraphVar.

8. The frequency of triads notation should be written as subscripts in section 2.6.2 Triadic interactions and their metrics and also in other places to be consistent.

In the new version of manuscript, this comment has been applied.

9. Figure 1 caption is in italics change it to normal font style. Also, the caption can be more specific to what the figure attempts to illustrate.

In the new version of manuscript, this comment has been applied.

[The Simple representation of frequency concept in graph domain. In this domain, the changes of signal across connected vertices define frequency levels (in time domain, the changes of signal across time points define frequency levels). Therefore, by moving from the lower frequency level to the higher frequency level of graph, the changes of signal across connected vertices are increased. Blue circles, red and blue lines are vertices, edges, and signals, respectively.] [lines 1-5 of page 10]

10. The plot of energy distributions as log distribution for the ASD and TC at different frequency bands may provide a better representation of the energy.

The log distribution is usually used when there are many observations for studied metric. In this study, the numbers of Un values were 52 and 44 for ASD and TC, respectively. As a result, a particular pattern or better observation did not attain. However, we have implemented your comment and the corresponding figure has been reported in Supplementary file.

[The Log-Log distribution of Un at LFB, MFB, and HFB are plotted in S3 Fig of S1_File.] [lines 8-9 of page 19]
